# Effect of ibuprofen on amelogenesis in Wistar rats

**DOI:** 10.1590/1678-7757-2024-0300

**Published:** 2025-05-23

**Authors:** Sharon OYHANART, Carlos A. BARCENAS, Ana Maria COLLET, Andrea Edith KAPLAN, Patricia Monica MANDALUNIS

**Affiliations:** 1 Universidad de Buenos Aires Facultad de Odontología Cátedra de Histología y Embriología Buenos Aires Argentina Universidad de Buenos Aires, Facultad de Odontología, Cátedra de Histología y Embriología, Buenos Aires, Argentina.; 2 Universidad de Buenos Aires Facultad de Odontología Cátedra de Anatomia Patologica Buenos Aires Argentina Universidad de Buenos Aires, Facultad de Odontologia, Cátedra de Anatomia Patologica, Buenos Aires, Argentina.; 3 Universidad de Buenos Aires Facultad de Odontología Cátedra de Materiales Dentales Buenos Aires Argentina Universidad de Buenos Aires, Facultad de Odontología, Cátedra de Materiales Dentales, Buenos Aires, Argentina.

**Keywords:** Molar hypomineralization, Animal Model, Ibuprofen, Amelogenesis

## Abstract

This work aimed to evaluate the effect of ibuprofen on amelogenesis using a model of continuously growing incisors in Wistar rats.

**Methodology:**

A total of eight female Wistar rats at weaning age were assigned to one of two groups (IBU and control). They were administered an 80 mg/kg dose of ibuprofen or an equivalent volume of distilled water for three weeks, then euthanized on day 16 of the experiment. Right hemimandibles were used to assess the mineral density of enamel using microtomography. The left hemimandibles were decalcified and processed to obtain sections, stained with Hematoxylin Eosin or immunohistochemical detection of amelogenin. Based on photomicrographs of hemimandibles, ameloblast and papillary layer height of the enamel and enamel organic matrix width were determined. The percentage of positive amelogenin was determined in immunohistochemically processed sections. Results were analyzed using Student’s t test.

**Results:**

IBU-treated animals showed lower body weight gain throughout the experiment (p<0.05). Mineral density and enamel thickness showed no significant differences. No significant differences in the height of the papillary layer or the width of the organic matrix were observed. Amelogenin expression in the ameloblast layer was lower in the experimental group. No significant difference was found between groups.

**Conclusion:**

The results obtained in this study model suggest that ibuprofen itself might not alter the amelogenesis process.

## Graphical abstract

**Figure:**
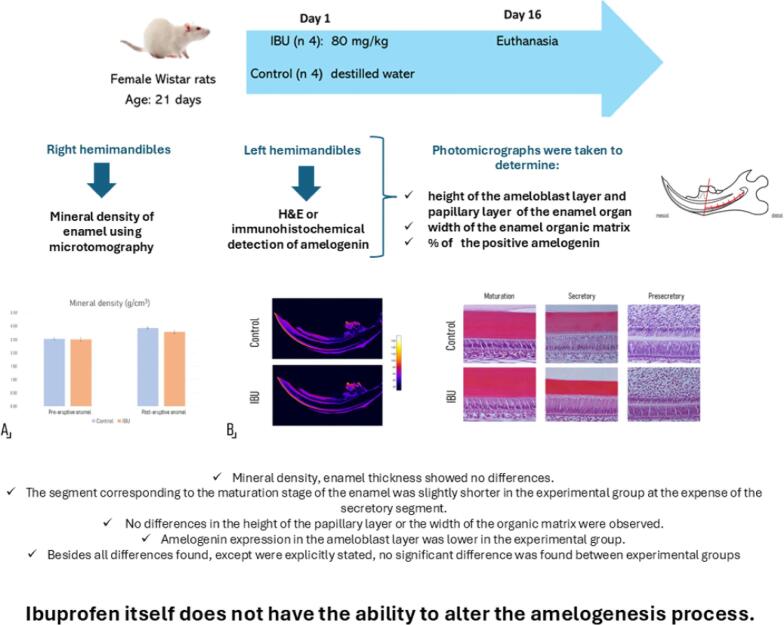

## Introduction

The term Molar Incisor Hypomineralization (MIH)^1^ has long been used to describe demarcated opacities on the dental enamel impacting one or several permanent first molars and may involve incisors. Nevertheless, despite the non-negligible 13.5% prevalence of MIH as reported by a systematic review^2^ and considerable efforts to shed light on this condition, its etiology remains unclear.

A recent systematic review and meta-analysis found that MIH was associated with conditions ranging from hypoxia and prematurity to respiratory ailments like such as and ruled out other factors previously considered, such as low body weight and maternal habits like smoking and consumption of different medications.^3^ However, most studies used data from questionnaires completed by caregivers, which can introduce a bias into the data because the tool relies on the caregivers’ memory to recall details of the patient’s clinical record. Thus, extrapolating conclusions in the search for a causal factor based on available data is difficult, and experimental models become highly relevant to study the alterations of amelogenesis, including MHI.

The process of amelogenesis is a highly orchestrated process at the cellular, molecular, and biochemical level triggered by epithelial-mesenchymal interactions.^4^ Largely, amelogenesis involves secretion of a matrix rich in proteins, mainly amelogenins, which are later resorbed to allow for the mineralization and growth of hydroxyapatite crystals and consolidation of the rods during the maturation phase.^5^ Failure of mature ameloblasts to resorb these proteins prevents crystal growth and leads to qualitative enamel defects in which the tissue has normal thickness but decreased mineral density.^6^ MIH is considered to result from an alteration in the maturation phase of ameloblasts.^6,7^

Experimental studies have shown alterations in amelogenesis associated with fluorides^8^and endocrine disruptors such as bisphenol A.^9,10^ Other reports have provided contradictory experimental evidence regarding pharmacological agents, such as amoxicillin—the most frequently explored drug in retrospective clinical studies on MIH.^11-13^

Recent findings suggest that fever during the first three years of life could be a predisposing factor for the onset of hypomineralization lesions.^3^ Even though the predisposing factor could be the drug used to manage the fever rather than the symptom itself, other non-steroidal anti-inflammatory agents have practically not been investigated.^14^

Ibuprofen (IBU) is one of the most frequently used non-steroidal anti-inflammatory drugs (NSAIDs) in pediatrics on account of its analgesic, anti-inflammatory, and antipyretic effect through the inhibition of prostaglandin synthesis by blocking the enzyme cyclooxygenase (COX-2). When orally administered, IBU is rapidly absorbed mainly from the gut and excreted through the kidney after being metabolized in the liver by cytochrome P450.^15^ Under the null hypothesis stating that ibuprofen has no effect on the amelogenesis process, the aim of this work was to evaluate the effect of ibuprofen on amelogenesis using a model of continuously growing incisors in Wistar rats.

## Methodology

### Animals and experimental design

In total, eight female Wistar rats at weaning age (21 days) were included. The animals were kept in groups of four per cage under 12:12 h light/dark cycles at 21±2°C and 52-56% relative humidity and allowed access to food (Cooperación standard chow; Buenos Aires, Argentina) and water *ad libitum*. All experimental procedures were performed in a controlled environment, following the eighth edition of the National Research Council Guide for the Care and Use of Laboratory Animals and were approved by the Institutional Committee for the Care and Use of Laboratory Animals of the School of Dentistry of the University of Buenos Aires (N° 009/2019 CICUAL-ODON-FOUBA Res. (CD) No. 330/19-01).The number of animals assigned to this study was decided considering ethical motives given the CICUAL-ODON-FOUBA recommendations to use the minimum possible number of animals per experiment.

The animals were randomly assigned to one of two identical groups of four (IBU and Control) and administered 80 mg of IBU per kg of body weight (Febratic 4%; Roemmers, Buenos Aires, Argentina) or an equal volume of distilled water (controls) through a gastric tube for three weeks. Allometric scaling for the dose was performed considering body surface area (BSA) and according to:

Animal equivalent dose (mg/kg) = Human dose (mg/kg) x (Km human/Km rat) in which the human dose was 13 mg/kg and the Km for a 1-year-old child (21.93) was estimated considering a weight of 10 kg and a BSA of 0.456 m2, based on the Monsteller formula, whereas the Km for the rats (3.42) was estimated from the mean weight of the animals allocated to the experimental group at the beginning of the experiment (i.e. 39 g). A BSA of 0.0111 m2 was estimated from Meeh’s formula. The dose of choice falls within the maximum daily safe dose for children (40 mg/kg), and although it would be normally split into different takes for pediatric patients, in our experimental design, animals were only given a single daily administration.

The animals were weighed daily throughout the 15 days of the experiment and euthanized on day 16 using a combination of 50 mg/kg of ketamin (Ketamin 50 - 50 mg/ml; Holliday-Scott S.A., Beccar, Buenos Aires, Argentina) and 5 mg/kg of xylazine (Xylazine 20 – 20 mg/kg; Richmond S.A., Buenos Aires, Argentina), followed by a lethal injection of 40 mg/kg of pentobarbital sodium and 5 mg/kg of phenytoin sodium (Euthanyle; Brouwer S.A., Buenos Aires, Argentina). The hemimandibles, tibiae, liver, kidneys, and gut were collected. The hemimandibles were fixed in buffered formalin at 4ºC for 48 hours.

### MicroCT

For determination of enamel mineral density in the continuously growing incisor, the right hemimandibles were fixed in buffered formalin for 48 h and then kept in 0.1% of sodium azide at 4°C until evaluation using a high resolution microtomography scanner (SkyScan 1272, Bruker, Kontich, Belgium) operated at 90 kV and 110 µA using a 0.5 mm Al filter. Each sample, two from the experimental group and two from the control group, was wrapped in tissue soaked in saline solution to prevent dehydration during scanning and placed in a plastic tube together with the calibration phantoms of known hydroxyapatite concentrations (0.25 and 0.75 g.cm^-3^/cm^3^) (Bruker, Kontich, Belgium). Flat-field correction was performed before scanning. The samples were rotated over 180°, and images were captured at 0.4° intervals taking three captures per position at a pixel size of 13 µm.

The projections were reconstructed using NRecon v 1.7.3.2 software (Bruker), applying corrections for artifacts produced by beam-hard tissue interactions (BHC: 7%; RAC: 5). The reconstructions were reoriented using DataViewer v1.5.6.2 software (Bruker) to place all the samples following the same anatomical landmarks in the three planes of space. The 8-bit images were analyzed using ImageJ software (National Institutes of Health, Bethesda, MD, USA) with a two-point linear calibration with respect to the hydroxyapatite phantoms mentioned above.

Enamel mineral density of the continuously growing incisor was assessed in 200 consecutive slices of pre-eruptive enamel and 200 slices of post-eruption enamel using an automated analysis macro.^16^ Enamel thickness at the limit between both types of enamel, defined as the point where the bone tissue formed a complete circumference around the incisor, was also measured.

### Histology (H-E) and Histomorphometry

After fixation in buffered formalin, the hemimandibles were decalcified in 10% EDTA at room temperature or at 4°, respectively. The samples were then processed histologically to obtain oriented H-E stained sections or for immunohistochemical detection of amelogenin as explained under the corresponding heading.

**Figure 1:**
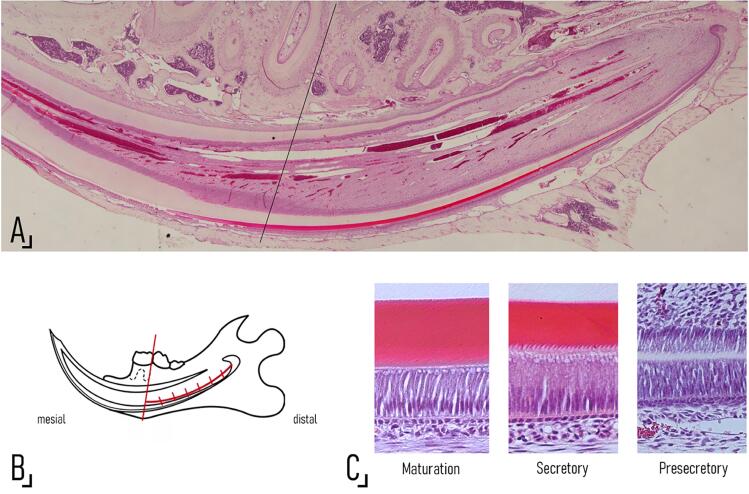
Photomicrographs of a sagittal H-E stained hemimandible section taken at 20× magnification and mounted using Photoshop software to obtain an image of the entire lower continuously growing incisor. The reference line coincides with the distal wall of the alveolus of the distal root of the first lower molar and is the anterior limit of the area selected to perform the histomorphometric determinations. The landmarks are shown in: B. A schematic drawing of the hemimandible of a Wistar rat. The vertical lines drawn through the curve at right angles and 1000 µm apart until the cervical loop represent the areas that were photomicrographed at 200× to perform histomorphometric determinations of the ameloblast and papillary layers of the enamel organ (further details are provided in the Materials and Methods section). C. Photomicrographs showing the three stages of enamel formation identified according to their histological features as seen under light microscopy (further details are provided in the Materials and Methods section).

Photomicrographs of sagittal Hematoxylin Eosin (HE) stained hemimandible sections taken at 20× magnification were mounted using Photoshop software to obtain a complete image of the incisor of each experimental unit (Figure 1). To standardize the histomorphometric determinations, all measurements were performed in an area of the incisor delimited by the cervical loop and the distal wall of the alveolus of the distal root of the first lower molar (Figure 1).

The obtained images were analyzed using ImagePro Plus to measure the distance between the landmarks mentioned above following the curvature of the incisor, and a microphotograph was taken every 1000 µm at a magnification of 200× (Figure 1). The following parameters were measured on the photomicrographs:

- Height of the ameloblast layer (Am.Ht; µm)
- Height of the papillary layer of the enamel organ (PaL.Ht; µm)
- Enamel matrix width (EnaM.Wi; µm)

Moreover, considering that the stages of amelogenesis in the continuously growing incisor of rodents are present in a spatially determined sequence along the entire length of the tooth, we sought to determine histologically whether ibuprofen administration caused a delay in enamel maturation. To this end, we identified the histological features that allow demarcating three stages that could be morphologically distinguished under light microscopy: the presence of enamel organic matrix as the transition between the pre-secretory and secretory-stages, and the absence of the Tome’s process as the transition between the secretory and maturation stages. As the landmarks were identified on each slide during direct microscopic examination, they were marked on the corresponding Photoshop image, and the length of the segment corresponding to each stage (maturation: MS.Le, secretory: SS.Le, pre-secretory: Pre.SSLe) was measured using ImagePro Plus.

### Immunohistochemical detection of amelogenin

The sagittal sections were deparaffinized and rehydrated in increasing concentrations of ethanol up to 70°, and the assay for detection of amelogenin was used. For antigen retrieval, the samples were incubated in 0.1% trypsin with tris-buffer (pH 7) at 37° for 10 min, after which endogenous peroxidase was blocked with 0.3% H_2_O_2_ in methanol. After rinsing in PBS, unspecific binding was blocked with normal serum (PK7800 R.T.U., Vectastain detection kit) for 25 min, and the primary anti-amelogenin antibody (1:600) (Santa Cruz Biotech) was applied. Revelation was performed using diaminobenzidine as chromogen (DAB substrate kit for peroxidase, SK4100, Vector Laboratories, Burlingame, CA, USA). Finally, the sections were dehydrated and mounted on Canada balsam. The primary antibody was omitted as negative control, and samples previously shown to be positive were used as positive control.

The obtained sections were processed following the same method used for HE stained sections, and photomicrographs were obtained at a magnification of 400× every 1000 µm along the entire length of the continuously growing incisor. The percentage of the positive stained area (%) was measured using ImagePro Plus, defining the total surface as the surface containing ameloblasts.

### Statistical analysis

Shapiro-Wilks was used to test the normality of data distribution, and the F Snedecor test was used to verify the homogeneity of variances. Because the data fulfilled both assumptions, Student’s *t* test was applied to compare both groups; for histomorphometric measurements performed along the incisor, each segment—numbered 1 to 7—was considered individually and the experimental and control groups compared; statistical significance was set at p<0.05, and analysis was performed using InfoStat v202 software.

## Results

### Body weight

**Figure 2:**
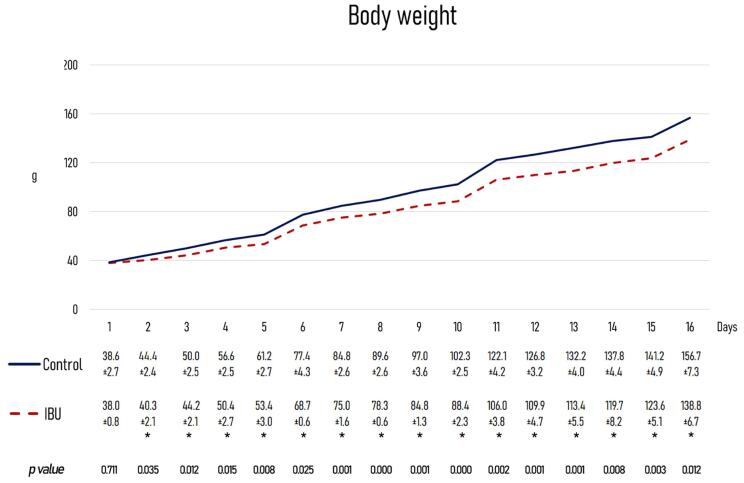
Weight curves. Body weight gain was significantly lower in experimental animals than in controls after the second day of the experiment. * *p*<0.05 between the control and experimental groups using Student’s t test.

Body weight gain was lower in animals treated with 4% ibuprofen throughout the experiment; the difference when compared to controls reached statistical significance after day two of treatment (Figure 2).

### Determinations using microCT

**Figure 3:**
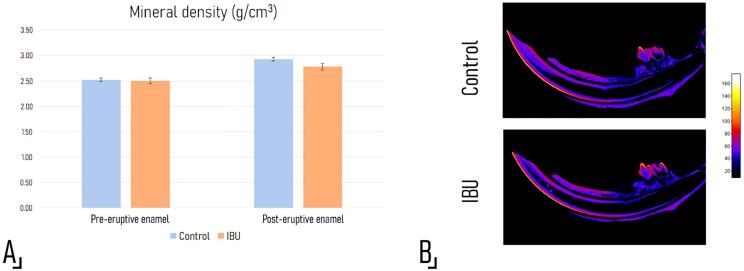
Bone mineral density assessment using microcomputed tomography scanning. A. Pre- and post-eruptive enamel mineral density values obtained by MicroCT are shown. No significant differences were observed between groups (p=0.59). B. Color maps of mineral density reflecting variations along the enamel from the incisal edge toward the area corresponding to the secretory stage, where values are similar to those of dentin, and it is not possible to distinguish between both tissues. No differences were observed between groups using Student’s t test, and there was no delay in the mineralization process as shown by the absence of an incisal displacement of the color gradient.

Mineral density assessment using high resolution microtomography showed no significant differences (p=0.59) at the level of pre-eruptive or post-eruptive enamel between the experimental (2.64±0.06 g.cm^3^) and the control group (2.67±0.01 g.cm^3^) (Figure 3). In addition, microCt showed no significant difference in enamel thickness (Control: 121.1±5.6, IBU: 122.4±3.1 µm, p=0.81).

### Histomorphometry of the enamel organ of the continuously growing incisor (HE)

**Figure 4:**
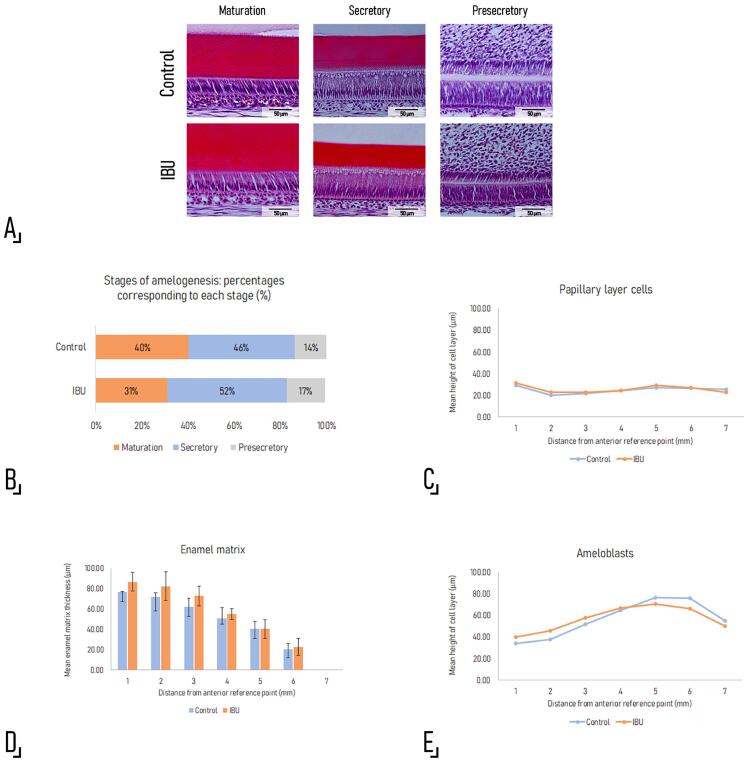
Histomorphometric evaluation of the amelogenesis process. A. Representative photomicrographs of sagittal H-E stained sections of a continuously growing incisor of an experimental and a control rat, showing the areas selected for histomorphometric analysis. No histological alterations were detected in any of the studied stages. Original magnification 200×. B. The graph shows the relative position of the areas corresponding to each stage (maturation, secretory, and pre-secretory) and the percentage of the total length corresponding to each stage, from the edge of the previous stage to the cervical loop. Although the transition between the secretory and the maturation stages was slightly displaced incisally in ibuprofen-treated animals, the difference between groups was not statistically significant (MS.Le: Control: 3.30±0.36 mm vs. IBU: 2.57±0.47 mm, p=0.07; SS.Le: Control: 3.83±0.54 mm vs.IBU: 4.27±0.06 mm, p=0.23). C. Height of the papillary layer of the enamel organ [PaL.H: p= 0.48 (1), 0.11 (2), 0.44 (3), 0.94 (4), 0.58 (5), 0.91 (6), 0.22 (7)]. D. Width of the enamel matrix [EnaM.W: p= 0.27 (1), 0.20 (2), 0.18 (3), 0.56 (4), 0.86 (5), 0.72 (6), 0.84 (7)]. E. Height of the ameloblast layer [Am Ht: p= 0.03 (1), 0.33 (2), 0.22 (3), 0.84 (4), 0.64 (5), 0.30 (6), 0.47 (7)]. The histomorphometric determinations correspond to measurements on photomicrographs taken every 1000 µm, from the previous landmark to the cervical loop, as shown in the schematic drawing in Fig. 1. Student’s t test was applied for all measurements and the numbers between brackets presented alongside the p-values represent each of the 7 segments at which the measurements were taken and compared between groups.

Histological examination revealed no morphologic alterations in the enamel organ or in the enamel matrix of the continuously growing incisor (Figure 4A).

The length of the segment corresponding to the maturation stage was slightly shorter in the experimental group (MS.Le: Control: 3.30±0.36 mm vs. IBU: 2.57±0.47 mm, p=0.07) at the expense of an increase in the length of the secretory segment (SS.L Control: 3.83±0.54 mm vs. IBU: 4.27±0.06 mm, p=0.23); the difference was also observed when the length of each segment was expressed as a percentage of the total length of the incisor of each experimental unit (MS.Le: Control: 40% vs. IBU: 31%, p=0.08; SS.Le: Control: 46% vs. IBU: 52%, p=0.11). As a result, the transition between both stages was incisally displaced, though the difference between groups was not statistically significant (MS.Le: p=0.07; SS.Le p=0.23) (Figure 4B).

No significant differences in the height of the papillary layer of the enamel organ [PaL.H (µm)] (Figure 4C), the thickness of the enamel matrix [EnaM.Wi (µm)] (Figure 4D), or the height of the ameloblast layer [Am Ht (µm)] were observed between groups.

### Amelogenin expression by immunohistochemical assays

Amelogenin expression in the ameloblast layer was lower in the experimental group (13±4%) than in the control group (27±9%), though the difference did not reach statistical significance (p=0.12).

## Discussion

In the last decades, there has been increasing information regarding structural, mechanical, and chemical alterations in MIH-affected enamel.^7^ Nevertheless, as mentioned earlier, the etiology of this condition remains unclear. According to recent clinical reports, fever in infancy could predispose to the presence of MIH lesions.^3^ Of note, most of the studies are retrospective^6,18^ and have a high risk of bias^3^, and therefore do not allow determining whether the cause of MIH lies in the symptom itself or in the mediation used for treatment. According to several pediatric associations and governmental organizations,^17,19^ ibuprofen,^20^ and paracetamol are the drugs of choice to relieve the discomfort of a febrile child. Free access to ibuprofen added to parental misconceptions about fever may lead to excessive drug use.^21^ Our literature review identified only one study on the impact of NSAIDs on enamel formation. The authors found that paracetamol caused a significant decrease in calcium and phosphorus content, as measured by X-ray energy dispersive spectroscopy (SEM-EDS), and in the immunohistochemical expression of cyclooxygenase 2 (COX-2) in the rat incisor, but they observed no alterations in response to ibuprofen.^14^ Importantly, the dose used in the study was 2.5 mg/day, which represents a human equivalent dose (HED) far lower than the usual pediatric dose.

Though higher than the dose recommended for use in rats (15 mg/kg),^22^ the ibuprofen dose used in this study (80 mg/kg) is eight times lower than the Lethal Dose 50% (LD_50_ ) (636 mg/kg) and is equivalent to a 13 mg/kg^23^ dose in humans, which is within the maximum-minimum dose limits recommended in pediatrics.^19^ Even though this study was performed using laboratory animals, the results obtained with this animal model might have qualitative validity for humans. Nevertheless, the experimental animals had lower body weight than controls throughout the entire course of the experimental period. This suggests that despite its good tolerability, administration of ibuprofen within the therapeutic range could cause some sort of gastro-intestinal irritation even though we could not observe histological alterations capable of explaining the lower weight gain (data not shown). A previous study^24^on the chronic toxicity of ibuprofen showed a decrease in body weight when the administered doses were substantially higher (180 mg/kg) than the one employed in this study; however, the authors only detected histological alterations in the gut in less than half the animals. Another study^25^ found far lower doses (15 mg/kg) capable of leading to alterations in body weight and peptic ulcers; in line with our results, the decrease in body weight occurred a few days after the onset of the study. On the other hand, studies employing the same dose as the one used here have reported significant histological alterations in the gut with no drop in the weight gain curve when compared to control animals.^26^ All in all, findings reported in experimental studies regarding the potential for gastrointestinal injury of ibuprofen are conflicting and vary greatly in the dose and administration protocols.^24-27^

The similarities between murines and humans in the stages of enamel formation^28,29^ have made rats and mice the most widely used animal models for studying enamel formation and its possible alterations.^30^ Conveniently, the continuously growing incisor of murines enables the analysis of all the stages of amelogenesis, which are present in a spatially determined sequence from the apical edge, where there is constant proliferation of more undifferentiated cells, to the tip, where attrition compensates for the continuous growth.^28^ This model has been used to evaluate the effect of different pharmacological drugs and compounds, such as fluorides,^31^ amoxicillin,^12^ and bisphenol A^9^ on amelogenesis. However, the parameters analyzed in the present study had not previously been used to assess the effect of NSAIDs. The average eruption rate of a lower incisor is 0.4 mm per day,^32^ therefore the total eruption throughout the duration of our experiment was 6 mm. Hence, the choice of this model and the duration of treatment were suitable for detecting histologic alterations caused by the action of ibuprofen on any of the stages of amelogenesis, including the maturation stage, which has been posited to be involved in MIH.^6^ Interestingly, we detected no histomorphometric alterations in any of these stages, suggesting that the dose of ibuprofen equivalent to the therapeutic range used in pediatrics would not affect the processes of enamel secretion and mineralization. Furthermore, although considered rather high for experimental animals, and despite resulting in changes in weight in the experimental group, the dose explored in the present study did not trigger deleterious effects upon enamel formation or mineralization. It could thus be expected that lower doses should not impair or affect amelogenesis negatively.

Despite the usefulness of microscopy in studying the phenomenon of amelogenesis, assessing the mineral content of enamel is essential to confirm the presence of hypomineralization. For this purpose, we used computed microtomography, a non-destructive high-resolution method that allows measuring mineral density based on the attenuation of the radiation as it passes through an object.^33^ Confirming our histomorphometric findings, MicroCT revealed no alterations in enamel mineral density or changes in enamel thickness after ibuprofen treatment. Microtomography has been shown to provide reliable results that correspond with data obtained using conventional and invasive methods for assessing mineral density such as ashing and backscattered electron microscopy (BSE).^34^ Nevertheless, its use to assess very low-density enamel, as would be the case of MIH, is limited due to its similarity to dentine.

Amelogenins account for over 90% of enamel proteins and play a central role in enamel formation, regulating hydroxyapatite crystal growth.^35^ Their removal during the maturation stage is a prerequisite for complete enamel mineralization.^36^ It has been shown that compounds like fluorides inhibit hydrolysis of these proteins in the enamel maturation stage,^37^ resulting in the presence of hypomineralized areas. Although amelogenins can be detected in the maturation stage,^38,39^ their expression is more intense in the secretory stage and decreases incisally. The immunohistochemical expression patterns observed here are in line with reports in the literature. However, when compared with our other findings, we observed no significant alterations associated with ibuprofen administration, which suggests that IBU might not have a direct effect on enamel mineralization. The amelogenin immunohistochemical positive staining measured in our study showed a reduction of expression close to 50% in IBU compared to control values. However, the statistical analysis showed no significant difference even though the standard deviation was within reasonable values. These results are probably due to the low number of animals per group.

MIH continues to pose a challenge in clinical practice, and the debate on its etiology is still relevant. Several authors suggest that besides systemic and environmental causes, a possible genetic predisposition cannot be ruled out.^40,41^ Although this issue is controversial,^42^ it has recently been posited that MIH is a condition of multifactorial origin and complex etiology and may be the result of epigenetic interactions.

Although the results obtained using this experimental model showed that ibuprofen by itself did not alter the amelogenesis process, it is worth noting that the reduced sample size represents a limitation. As previously mentioned, efforts were made to keep it to a minimum for ethical reasons. Another possible limitation is the choice of dose, as it may have been relatively high for animals of this weight and age. However, it was estimated considering the maximum daily dose considered safe for pediatric use; instead of splitting it into multiple administrations, it was given once daily in our experimental design.

Considering the results obtained in this experimental model, the null hypothesis was accepted, as ibuprofen by itself did not alter the amelogenesis process.
